# The Predictors of Incidental Durotomy in Patients Undergoing Pedicle Subtraction Osteotomy for the Correction of Adult Spinal Deformity

**DOI:** 10.3390/jcm13020340

**Published:** 2024-01-07

**Authors:** Abdelrahman M. Hamouda, Zach Pennington, Maria Astudillo Potes, Anthony L. Mikula, Nikita Lakomkin, Michael L. Martini, Kingsley O. Abode-Iyamah, Brett A. Freedman, Jamal McClendon, Ahmad N. Nassr, Arjun S. Sebastian, Jeremy L. Fogelson, Benjamin D. Elder

**Affiliations:** 1 Department of Neurologic Surgery, Mayo Clinic, Rochester, MN 55905, USA; hamouda.abdelrahman@mayo.edu (A.M.H.); astudillo.maria@mayo.edu (M.A.P.);; 2 Department of Neurologic Surgery, Mayo Clinic, Jacksonville, FL 32224, USA; 3 Department of Orthopaedic Surgery, Mayo Clinic, Rochester, MN 55905, USA; 4 Department of Neurologic Surgery, Mayo Clinic, Phoenix, AZ 85054, USA; mcclendon.jamal@mayo.edu

**Keywords:** pedicle subtraction osteotomy, complication, adult spinal deformity, revision surgery

## Abstract

**Background**: Pedicle subtraction osteotomy (PSO) is a powerful tool for sagittal plane correction in patients with rigid adult spinal deformity (ASD); however, it is associated with high intraoperative blood loss and the increased risk of durotomy. The objective of the present study was to identify intraoperative techniques and baseline patient factors capable of predicting intraoperative durotomy. **Methods**: A tri-institutional database was retrospectively queried for all patients who underwent PSO for ASD. Data on baseline comorbidities, surgical history, surgeon characteristics and intraoperative maneuvers were gathered. PSO aggressiveness was defined as conventional (Schwab 3 PSO) or an extended PSO (Schwab type 4). The primary outcome of the study was the occurrence of durotomy intraoperatively. Univariable analyses were performed with Mann–Whitney U tests, Chi-squared analyses, and Fisher’s exact tests. Statistical significance was defined by *p* < 0.05. **Results**: One hundred and sixteen patients were identified (mean age 61.9 ± 12.6 yr; 44.8% male), of whom 51 (44.0%) experienced intraoperative durotomy. There were no significant differences in baseline comorbidities between those who did and did not experience durotomy, with the exception that baseline weight and body mass index were higher in patients who did not suffer durotomy. Prior surgery (OR 2.73; 95% CI [1.13, 6.58]; *p* = 0.03) and, more specifically, prior decompression at the PSO level (OR 4.23; 95% CI [1.92, 9.34]; *p* < 0.001) was predictive of durotomy. A comparison of surgeon training showed no statistically significant difference in durotomy rate between fellowship and non-fellowship trained surgeons, or between orthopedic surgeons and neurosurgeons. The PSO level, PSO aggressiveness, the presence of stenosis at the PSO level, nor the surgical instrument used predicted the odds of durotomy occurrence. Those experiencing durotomy had similar hospitalization durations, rates of reoperation and rates of nonroutine discharge. **Conclusions**: In this large multisite series, a history of prior decompression at the PSO level was associated with a four-fold increase in intraoperative durotomy risk. Notably the use of extended (versus) standard PSO, surgical technique, nor baseline patient characteristics predicted durotomy. Durotomies occurred in 44% of patients and may prolong operative times. Additional prospective investigations are merited.

## 1. Introduction

Adult spinal deformity (ASD) is represented by sagittal and/or coronal plane imbalance, and is common in patients with degenerative spinal pathology [1]. It has been reported to affect 6% of adults older than 50 years of age [1], though some reports, notably that of Schwab et al. [2] have reported radiographic scoliosis to affect upwards of two-thirds of patients over 60 years of age. ASD has a significant negative impact on the mental and physical well-being of affected patients, with prior quality-of-life (QoL) studies suggesting sagittal imbalance is correlated with the overall degree of disability [3]. To this end, Pellisé et al. [4] noted that the negative impact of ASD on reported QoL exceeds that of other chronic conditions, including diabetes, congestive heart failure and chronic lung disease, and is greatest in those requiring surgical intervention. Among these surgical patients, intervention often involves multilevel posterior instrumented fusion with interbody placement and osteotomies to reduce the deformity [5].

A nontrivial proportion of patients experience mechanical complications, including proximal junctional kyphosis (PJK) [6], that require surgical revision. Additionally, some patients initially undergo a less invasive focal treatment, designed to treat radicular pain due to foraminal stenosis within the kyphoscoliotic deformity, but subsequently require definitive management. Such revision operations have an increased risk for durotomy [7], and may require aggressive bony work, including a three-column (3CO) or pedicle subtraction osteotomy (PSO) to achieve sagittal and/or coronal plane correction, particularly in a previously fused spine [8]. Such aggressive correction has a high associated morbidity, with work from the Scoliosis Research Society [9] noting complications in nearly a third of patients. One complication—durotomy—occurred in 6% of cases, and can lead to meningitis due to the free communication between the wound and the intrathecal space and complicated drain management and wound healing, and can potentially inhibit wound healing by inhibiting local angiogenesis [10]. Incidental durotomy, therefore, has a significant potentially negative impact on ASD surgeries involving pedicle subtraction osteotomy. The objective of the present study is to identify those risk factors for incidental durotomy in patients undergoing surgical correction of ASD with PSO.

## 2. Materials and Methods

After obtaining IRB approval, a single institution database comprising three tertiary care centers was queried for all patients who underwent 3CO as part of an instrumented spine procedure between February 2008 and February 2023. All patients treated at these centers were prospectively enrolled in the registry as a part of an ongoing quality improvement effort. The registry can be searched based upon patient demographics, procedural codes and diagnostic codes, among other details pulled directly from the electronic medical record. In the present study, the database was queried by diagnosis with the search terms “adult spinal deformity” or “scoliosis”, and the procedural terms “pedicle subtraction osteotomy” and “spinal fusion” or “lumbar fusion” or “thoracolumbar fusion”. After identifying potential patients from the registry, patient records were individually queried to ensure that surgery was performed for ASD, as defined by the Schwab criteria: coronal Cobb angle ≥ 20°, sagittal vertical axis (SVA) ≥ 5 cm, thoracic kyphosis ≥ 60°, or pelvic tilt ≥ 25° [11]. Patients were included if: (1) they met radiographic criteria for adult spinal deformity, (2) underwent long-segment thoracolumbar instrumented fusion including one or more pedicle subtraction osteotomies and (3) had complete medical records including prior spine surgery history, operative notes and postoperative follow-up detailing the need for surgical revision. Patients were excluded if they: (1) were pediatric patients (<18 years of age), (2) underwent surgery for an indication other than adult spinal deformity (including infection, pathologic fracture, trauma, or malignancy) or (3) underwent sagittal plane correction with only a combination of Schwab 1 and Schwab 2 osteotomies (i.e., did not undergo a PSO or extended PSO).

For patients that met these criteria, details were collected on demographics (e.g., age, sex), medical comorbidities (including modified Frailty Index-5 item (mFI5) and Charlson comorbidity Index (CCI)), chronic (≥6-week) corticosteroid use, surgical history (index versus revision surgery), surgeon details (specialty, fellowship training, experience/years post-residency), operative details (construct length, type of osteotomy, osteotomy level), and durotomy details (where durotomy occurred). For this study, durotomy was defined as the intraoperative detection of a complete dural tear with cerebrospinal egress; partial thickness dural tears were not considered, given the argument by prior authors that repairing with sealant alone is sufficient for these defects [12]. Additionally, preoperative radiology reports were queried to identify whether the patient had documented radiographic stenosis at the level of the PSO (defined as radiologist report of moderate or severe stenosis).

### 2.1. Procedure Description

The details of pedicle subtraction osteotomy have been described in details by others, including this recent description by Gupta and colleagues [9]. In short, patients in this study all underwent open thoracolumbar instrumented fusion with a PSO or extended PSO. Midline incisions were made, and the target vertebral levels were exposed in subperiosteal fashion. Pedicle screws were placed using established landmarks (leaving out the planned PSO level). Laminectomy of the PSO level was performed, or, where prior decompression had been performed, the scar was debulked to prevent buckling of the dura with closure of the PSO. Complete bilateral facetectomies were then performed and the pedicles were taken down bilaterally. Temporary rods were placed across the planned PSO site to stabilize the spinal column during resection of the middle and anterior columns. If an extended PSO was performed, the diskectomy was completed prior to placement of the temporary rods. Using a nerve root retractor to protect the thecal sac, the osteotomy wedges were completed bilaterally using a combination of osteotomes, rongeurs, a high-speed drill, and an ultrasonic bone cutting device, as determined by surgeon preference. Once the PSO was completed, the rods were compressed across the PSO site and, one at a time, final rods were placed. The wound was then closed in standard fashion.

### 2.2. Statistical Analysis

Data were collected using Microsoft Excel Version 2207 (Build 15427.20308 Click-to-Run) (Redmond, WA, USA) and checked by three independent reviewers (AH, ZP, MAP). Data were summarized as mean ± standard deviation for continuous data and counts with percentages for discrete data. Statistical Analysis was performed using SPSS 28.0.0 (IBM, Armonk, NY, USA). Univariable comparisons between patients who did and did not experience durotomy were compared using Mann–Whitney U tests for continuous variables, Fisher’s exact tests for dichotomous variables and χ^2^ analyses for ordinal and categorical data. Those variables identified as significant at the *p* < 0.05 level on univariable analysis were entered into multivariable analysis to identify independent statistically significant predictors of durotomy. Results of the logistic regression were expressed as odds ratio (OR) with associated 95% confidence intervals (95% CI).

## 3. Results

A total of 116 patients were identified (mean age 61.9 ± 12.6 yr; 44.8% male), of whom 51 (44.0%) experienced intraoperative durotomy, though none resulted in new neurological deficits (Table 1). All patients underwent only a single PSO or extended PSO; none underwent more than one three-column osteotomy. A comparison of baseline characteristics between the “durotomy” and “no durotomy” groups showed no statistically significant differences with respect to age (*p* = 0.55), sex (*p* = 0.35), smoking history (*p* = 0.85), mFI5 (*p* = 0.21), CCI (*p* = 0.76) or chronic steroid use (*p* = 0.24). The sole exceptions were that patients not experiencing durotomy had a higher weight BMI (30.9 ± 5.5 vs. 28.5 ± 5.6 kg/m^2^; *p* = 0.04) than those experiencing durotomy.

An evaluation of surgeon characteristics (n = 18 unique surgeons) showed no statistically significant difference in the odds of intraoperative durotomy between patients treated by a neurosurgeon versus an orthopedic spine surgeon (*p* = 0.40) or a surgeon with fellowship training versus one without fellowship training (*p* = 0.99). A review of surgical details (Table 2) showed that the only significant predictors of intraoperative durotomy were having undergone prior thoracolumbar surgery, (82.4 vs. 63.1%; *p* = 0.02) and more specifically, having previously undergone a decompression procedure at the level of the PSO (72.5 vs. 38.5%; *p* = 0.001). There was no significant inter-group difference regarding the surgical tool(s) employed to perform the osteotomy, including use of the ultrasonic bone cutter (*p* = 0.17). Though, of note, the use of the ultrasonic bone cutter for osteotomy formation was less common in the durotomy group. The placement of an interbody device at the PSO level (*p* = 0.99), aggressiveness of the PSO—Schwab 3 vs. Schwab 4 PSO (*p* = 0.25), PSO level (*p* = 0.74), nor the presence of canal stenosis at the PSO level (*p* = 0.24) predicted durotomy risk. 

The bivariate logistic regression of durotomy odds as a function of surgical history showed that patients with a history of prior lumbar procedures had nearly three-fold higher odds of intraoperative durotomy (OR 2.73; 95% CI [1.14, 6.58]; *p* = 0.025). A history of prior decompression at the PSO level was even more closely associated with durotomy odds, portending a more than four-fold increase (OR 4.23; 95% CI [1.92, 9.34]; *p* < 0.001). On multivariable analysis, only having undergone a prior laminectomy at the planned PSO site was predictive of intraoperative durotomy (OR 4.12; 95% CI [1.41, 12.03]; *p* = 0.009); BMI (OR 0.93; 95% CI [0.87, 1.00]; *p* = 0.061) nor undergoing revision [vs index] thoracolumbar surgery alone (OR 0.93; 95% CI [0.28, 3.10]; *p* = 0.93) were predictive of durotomy.

Primary durotomy closure was achieved in 48 of 51 patients. The sutures’ materials included nylon (Nurolon™, Ethicon, Raritan, NJ, USA), polypropylene monofilament (Prolene™, Ethicon, Raritan, NJ, USA) and polytetrafluoroethylene (Gortex, Gore Medical, Flagstaff, AZ, USA)™. In 35 cases, a dural sealant was employed (32 employed sealant to reinforce the primary closure). Closures were bolstered with a muscle pledget in 22 patients, an acellular collagen graft (DuraGen^®^, Integra LifeSciences, Princeton, NJ, USA) in 11 cases, and gelatin foam in 4 cases. None of the patients underwent the placement of a lumbar or external ventricular drain. Six patients required reoperation for wound complications. Of the fifty-one cases of durotomy, two patients were symptomatic, both presenting with headaches. One patient was successfully treated with an epidural blood patch. The second patient was initially managed with an epidural blood patch, which was unsuccessful and required a return to the operating room for an ongoing CSF leak. The durotomy was originally repaired with a muscle pledget, polypropylene suture, and fibrin glue. The wound was reopened and repaired with a running 5-0 Prolene™ (Ethicon, Raritan, NJ, USA), a gelatin sponge onlay, and fibrin glue.

## 4. Discussion

Three-column osteotomies, including pedicle subtraction osteotomies (Schwab type 3 osteotomy) and extended pedicle subtraction osteotomies (Schwab type 4 osteotomy) [13], are associated with complications in approximately one-third of patients [14], even in the hands of experienced surgeons. One complication, durotomy, can result in impaired wound healing, requiring surgical revision and potentially persistent cerebrospinal fluid (CSF) leaks and meningitis in the setting of wound infections. In the present multicenter study, we examined 116 patients undergoing open corrections of ASD using one or more PSOs. Interestingly, we found that the only significant predictors of durotomy were prior lumbar surgery, and, more specifically, prior decompression procedure at the PSO level, which was associated with an over four-fold risk in the odds of durotomy. While BMI was also statistically higher in the “no durotomy” group, we feel this is most suggestive of sampling bias, given a recent meta-analysis by Alshameeri et al. [15] failed to find any significant association between obesity and the risk of intraoperative dural tear. Interestingly, neither surgeon training nor the need for extended PSO predicted the occurrence of durotomy. Of note, the intraoperative durotomy rate (44%) was high relative to rates quoted by studies examining general spine practices (7–10%) [7,16,17,18], though similar rates have been reported in other single-institution series exclusively examining adult spinal deformity surgery, including that of Chan et al. [19].

### 4.1. Prior Studies of Risk Factors for Durotomy in ASD Surgery

#### 4.1.1. Revision Surgery and Scarring

As in the present study, multiple other studies have suggested prior surgery portends an increased risk of intraoperative durotomy. Ehresman and colleagues [7] presented a retrospective cohort of 1279 patients who underwent thoracolumbar surgery, of whom 8.4% experienced durotomy. They noted that the independent risk factors for durotomy were a delayed surgical start, undergoing revision surgery, advanced patient age and increasing surgical time. In their series of 1430 lumbar surgeries (10% with durotomy), Baker and colleagues [13] similarly noted advanced patient age, revision surgery and increased surgical invasiveness as risk factors for durotomy, with revision surgery being the strongest individual risk factor. Herren and colleagues [14] subsequently reported revision surgery to be associated with 78% increased odds of dural tears in their multicenter series of 3254 lumbar surgeries, of which 328 (10.1%) were complicated by durotomy. Subsequently, Iyer et al. [17] described durotomy risk based upon 564 patients from the multicenter ISSG dataset. Durotomy occurred in 10.8% of cases. As in the present study, the authors reported a history of prior decompression to be the only significant risk factor for intraoperative durotomy. Of note, this earlier cohort had a lower prevalence of revision surgery (47.2%) compared to the present study (71.5% of patients), which may in part account for the observed difference in durotomy rates.

Increased durotomy risk in the context of revision surgery is likely related to epidural scarring, which has been noted by multiple prior groups [18]. In the context of ASD surgery requiring osteotomy for correction, Arlet [19] recommended performing the osteotomy away from the level of scarring. The present results support this as a potential protective maneuver, as the biggest predictor of durotomy in the present study was having previously undergone a decompression procedure at the PSO level. However, avoiding PSO execution at the prior laminectomy site may be infeasible, depending upon the level and location of the fixed deformity in revision cases. It may also place the lordosis in a non-physiological location, and thereby be an unfavorable strategy from the perspective of achieving alignment goals. In such cases, decompression should start from regions of normal anatomy and then move towards taking down the scar and exposing the landmarks for the osteotomy. The dural tube should be freed of all adhesions to the pedicles and vertebral body of the PSO level prior to initiating the osteotomy. Though clinical experience suggests it is during this step that the surgeon is at highest risk of durotomy, such maneuvers are necessary to create a working channel for the osteotomy tools and to prevent the excessive buckling of the dura during the closure of the PSO.

The above strategies have been described as potential methods for decreasing durotomy risk while performing a PSO, though there are no surefire techniques for preventing this outcome. In general, our strategy is the first to consider performing the PSO at a level other than the level of the prior decompression. This allows the surgeon to work with a preserved/normal anatomy and to avoid regions of epidural scarring, which the current literature suggests is likely a driver of durotomy risk. However, if the PSO must be performed at a level of prior decompression in order to achieve the necessary sagittal correction, then the dissection should start from a region of normal or relatively preserved anatomy. The scar should then be taken down piecemeal from the region of normal anatomy to that which is most abnormal. All scar material need not be removed if the combined scar/dura is pliant enough to tolerate compression across the PSO site without the buckling of the dura. During the decompression, the adhesions to the pedicles and vertebral body of the PSO level should be lysed, so that an incidental durotomy is not encountered during execution of the PSO. Such a tear will likely be very eccentric/lateral and likely not amenable to primary repair, increasing the potential risk of persistent CSF leaks.

#### 4.1.2. Surgeon Experience

There have been limited investigations into the influence of surgeon experience on durotomy risk. Recently, Winter and colleagues [20] examined a retrospective cohort of 650 patients who underwent lumbar surgery. They found that laminectomy versus sublaminar decompression increased the risk of durotomy, but neither surgeon experience nor revision (versus index) surgery predicted durotomy occurrence. By contrast, Enders et al. [21] published an earlier series of 541 patients who underwent lumbar interbody fusion over a 10-year period, finding that inexperienced surgeons (defined as having performed <40 prior interbody fusions) were more than twice as likely to experience a durotomy than experienced surgeons (a minimum of 150 prior interbody fusions performed). Prior surgery (OR 2.63; *p* < 0.001) and multi-segment (versus single segment) surgery (OR 1.43; *p* = 0.03) were also risk factors for durotomy. Sin et al. [22] reported similar findings in their series of 76 patients, of whom 12 suffered durotomies. The authors reported that 75% of the durotomies were caused by residents in training, suggesting again that increased experience may reduce the risk of intraoperative durotomy. McMahon et al. [23] similarly noted residents to be responsible for the largest portion of durotomies, with fellows and attending surgeons accounting for far smaller proportions. Lastly, Raad and colleagues [24] reported on a single surgeon series of 197 PSOs. Though durotomy was not specifically examined, neurological injury—an often-related outcome—was examined and was noted to decrease with increased experience (estimated at 8% per 100 cases). This echoed earlier findings by Lau et al. in a 12-year cohort, where they found that increased years of surgeon operative experience was associated with a decreased risk of neurologic injury amongst patients undergoing 3CO for ASD. They noted a relative plateau after 3–5 years of independent operative experience.

#### 4.1.3. Schwab Grade of Osteotomy

To our knowledge, there has been no previous investigation of the association between osteotomy size and odds of durotomy. Previously, it has been reported that the risk of durotomy is increased in patients undergoing osteotomy for sagittal or coronal alignment correction. Using the ISSG database, Iyer et al. [17] reported that the performance of a PSO was associated with a 2.8-fold increased risk of durotomy, though this association was only significant in univariable analyses. There has been no such examination comparing risk following PSO, extended PSO, or vertebral column resection. Additionally, the data of Iyer et al. [17] showed a greater PI–LL mismatch and a more severe sagittal deformity (as measured by SVA) preoperatively, with no significant difference postoperatively, suggesting that sagittal plane correction may have been more aggressive in the durotomy group, though this was not formally examined.

#### 4.1.4. Ultrasonic Bone Curette and the Influence of Osteotomy Tools

The influence of the decompression instrument on durotomy risk has been only superficially examined, with most studies reporting Kerrison rongeur use to be associated with the occurrence of durotomy, though without formalized statistical examination [22,23]. Here, we sought to examine the influence of osteotomy tools (high-speed drill/burr, ultrasonic cutting instrument, osteotome, etc.) on the occurrence of durotomy. The ultrasonic cutting tool has been reported to have a decreased associated risk of durotomy in small series, including that of Bydon et al. [25] and Steinle et al. [26], though this association has been inconsistent [27]. Some authors [19] advocate against the use of this tool in the context of epidural scars, as they believe it actually increases the risk of durotomy. In the present series, we found that the performance of osteotomy cuts with an ultrasonic bone cutter was not associated with an increased risk of durotomy. In fact, though a non-significant finding due to the small sample size, the use of an ultrasonic cutting device was potentially protective of durotomy, as it was used nearly two-fold more commonly in the group that did not suffer durotomy (16.9 vs. 7.8%; *p* = 0.17).

### 4.2. Limitations

The present study has several limitations, including its reliance on retrospective data. This precludes us from reaching any conclusion about the potential causal linkage between the identified risk factors and durotomy occurrence. Additionally, the present study makes use of data from a single institution. Nevertheless, the data were gathered from three tertiary care sites staffed by surgeons trained at multiple distinct centers from both orthopedic and neurosurgical disciplines, so they help to improve the generalizability of the present results. An additional limitation is the potential for incompleteness in the operative notes. While many surgeons employ several tools during the execution of a PSO, all may not be named in their operative report, leading to potential under-reporting. It is therefore possible that there is an association between intraoperative durotomy and one of the osteotomy techniques, which was not captured due to incompleteness of the operative notes. Furthermore, while the use of a PSO versus an extended PSO was investigated as a risk factor for intraoperative durotomy, the classification was based upon surgeon reports of the procedure. An alternative radiographic method for assessing the osteotomy (i.e., the bony wedge angle) may be an aspect to explore in future investigation and may reveal a threshold for osteotomy “aggressiveness”, above which there is an elevated risk of durotomy. A final limitation is the relatively small sample size. While a multiyear, multicenter dataset was employed, the power of the results can be improved in further iterations through the inclusion of additional centers or study groups. Finally, prospective data collection could facilitate the determination of causal factors for durotomy, which may better identify potential points for therapeutic intervention.

## 5. Conclusions

In the present multicenter retrospective cohort study, a history of prior lumbar surgery was the only significant predictor of intraoperative durotomy during adult spinal deformity surgery incorporating one or more pedicle subtraction osteotomies. Specifically, a history of a prior decompressive procedure at the planned PSO site was the greatest risk factor, portending a more than four-fold increase in the odds of durotomy. Pedicle subtraction osteotomy size (Schwab 3 vs. 4) nor surgeon training successfully identified patients’ risk of durotomy. Additional investigations using prospectively gathered data are merited to validate these results.

## Figures and Tables

**Table 1:** Demographics of included patients and univariable comparisons for risk factors for intraoperative durotomy.

Variable		All Patients	Durotomy	No Durotomy	*p*
*Demographics*	N	116	51	65	
Age (yr)	116	61.9 ± 12.6	63.2 ± 11.1	60.9 ± 13.7	0.55
Sex (% male)	116	52 (44.8)	20 (39.2)	32 (49.2)	0.35
Race	116				0.49
White		112 (96.6)	49 (96.1)	63 (96.9)	
Black		3 (2.6)	1 (2.0)	2 (3.1)	
Other		1 (0.9)	1 (2.0)	0 (0)	
BMI (kg/m^2^)	116	29.8 ± 5.6	28.5 ± 5.6	30.9 ± 5.5	**0.04**
Smoking History	116				0.80
Current		14 (12.1)	5 (9.8)	9 (13.8)	
Former		38 (32.8)	17 (33.3)	21 (32.3)	
Never		64 (55.2)	29 (56.9)	35 (53.8)	
Any		52 (44.8)	22 (43.1)	30 (46.2)	0.85
*Medical Comorbidities*					
ASA	116	2.7 ± 0.6	2.7 ± 0.5	2.6 ± 0.6	0.27
*mFI5*	116	0.9 ± 0.9	0.8 ± 0.8	1.0 ± 0.9	0.21
Diabetes Mellitus	116	22 (19.0)	7 (13.7)	15 (23.1)	0.24
HTN Requiring Medication	116	64 (55.2)	23 (45.1)	41 (63.1)	0.06
Functional Dependence	116	12 (10.3)	8 (15.7)	4 (6.2)	0.13
COPD or Pneumonia	116	9 (7.8)	3 (5.9)	6 (9.2)	0.73
CHF Exacerbation in Past 30 d	116	1 (0.9)	0 (0)	1 (1.5)	0.99
*CCI*	116	1.0 ± 1.4	1.1 ± 1.4	1.0 ± 1.3	0.76
MI	116	3 (2.6)	1 (2.0)	2 (3.1)	0.99
CHF	116	7 (6.0)	4 (7.8)	3 (4.6)	0.70
PVD	116	8 (6.9)	2 (3.9)	6 (9.2)	0.46
CVA	116	3 (2.6)	1 (2.0)	2 (3.1)	0.99
Dementia	116	2 (1.7)	1 (2.0)	1 (1.5)	0.99
COPD	116	10 (8.6)	6 (11.8)	6 (9.2)	0.76
CTD	116	10 (8.6)	6 (11.8)	4 (6.2)	0.33
PUD	116	0 (0)	0 (0)	0 (0)	0.99
Liver disease	116				
Mild		5 (4.3)	0 (0)	5 (7.7)	0.07
Moderate/Severe		0 (0)	0 (0)	0 (0)	0.99
DM	116				
Without EOD		19 (16.4)	6 (11.8)	13 (20.0)	0.31
With EOD		3 (2.6)	1 (2.0)	2 (3.1)	0.99
Hemiplegia	116	2 (1.7)	2 (3.9)	0 (0)	0.19
Moderate/Severe CKD	116	12 (10.3)	5 (9.8)	7 (10.8)	0.99
Malignancy	116				
Without metastases		8 (6.9)	6 (11.8)	2 (3.1)	0.14
With metastases		0 (0)	0 (0)	0 (0)	0.99
Leukemia	116	1 (0.9)	0 (0)	1 (1.5)	0.99
Lymphoma	116	0 (0)	0 (0)	0 (0)	0.99
AIDS	116	0 (0)	0 (0)	0 (0)	0.99
≥6-week Steroid Use	116	7 (6.0)	5 (9.8)	2 (3.1)	0.24

AIDS—acquired immunodeficiency syndrome; ASA—American Society of Anesthesiologists; BMI—body mass index; CCI—Charlson Comorbidity Index; CHF—congestive heart failure; CKD—chronic kidney disease; COPD—chronic obstructive pulmonary disease; CTD—connective tissue disease; CVA—cerebrovascular accident; d—day; HTN—hypertension; kg—kilogram; m—meter; mFI—modified frailty index; MI—myocardial infarction; PUD—peptic ulcer disease; PVD—peripheral vascular disease; yr—year.

**Table 2:** Operative details for the included patients and univariable comparisons for risk factors for intraoperative durotomy.

Variable		All Patients	Durotomy	No Durotomy	*p*
*Surgical Details*					
Revision [vs Index]	116	83 (71.6)	42 (82.4)	41 (63.1)	**0.02**
Prior lami at PSO level	116	62 (53.4)	37 (72.5)	25 (38.5)	**<0.01**
Osteotomy Tool Used	116				
Curette		39 (33.6)	22 (43.1)	17 (26.2)	0.08
Drill		51 (44.0)	22 (43.1)	29 (44.6)	0.99
Rongeur		63 (54.3)	29 (56.9)	34 (52.3)	0.71
Ultrasonic Bone Cutter		15 (12.9)	4 (7.8)	11 (16.9)	0.17
Osteotome		95 (81.9)	42 (82.4)	53 (81.5)	0.99
Cage Placed at PSO Level	116	57 (49.1)	25 (49.0)	32 (49.2)	0.99
PSO Type	116				0.25
Schwab 3		69 (59.5)	27 (52.9)	42 (64.6)	
Extended PSO (Schwab 4–5)		47 (40.5)	24 (47.1)	23 (35.4)	
PSO Level	116				0.74
L1		6 (5.2)	3 (5.9)	3 (4.6)	
L2		16 (13.8)	6 (11.8)	10 (15.4)	
L3		35 (30.2)	14 (27.5)	21 (32.3)	
L4		49 (42.2)	25 (49.0)	24 (36.9)	
L5		9 (7.8)	3 (5.9)	6 (9.2)	
S1		1 (0.9)	0 (0)	1 (1.5)	
Stenosis at PSO Level	116				0.24
None		64 (55.2)	29 (56.9)	35 (53.8)	
Mild		28 (24.1)	15 (29.4)	13 (20.0)	
Moderate		13 (11.2)	5 (9.8)	8 (12.3)	
Severe		11 (9.5)	2 (3.9)	9 (13.8)	
Surgeon Specialty	116				0.40
Neurosurgery		86 (74.1)	40 (78.4)	46 (70.8)	
Orthopedic Surgery		30 (25.9	11 (21.6)	19 (29.2)	
Fellowship-Trained	116	105 (90.5)	46 (90.2)	59 (90.8)	0.99
*Outcomes*					
Length of Stay (d)	116	8.6 ± 5.8	9.5 ± 7.5	7.9 ± 3.9	0.67
Non-home Discharge	116	53 (45.7)	22 (43.1)	31 (47.7)	0.71

PSO—pedicle subtraction osteotomy.

## Data Availability

The data presented in this study are available on request from the corresponding author (accurately indicate status).
